# Supplementary material for: Regulated RIAM–Talin Engagement Controls Adhesion Stability and Mechanical Output
Source: bioRxiv. 2026 Jan 16:2026.01.15.699718. Preprint. [Version 1] doi: 10.64898/2026.01.15.699718 (PMC12871151; doi:10.64898/2026.01.15.699718)
Supplement: 1 [file NIHPP2026.01.15.699718V1-supplement-1.pdf]

bioRxiv preprint doi: https://doi.org/10.64898/2026.01.15.699718; this version posted January 16, 2026. The copyright holder for this preprint (which was not certified by peer review) is the author/funder, who has granted bioRxiv a license to display the preprint in perpetuity. It is made available under aCC-BY 4.0 International license.

Supplementary Figures

Figure S1

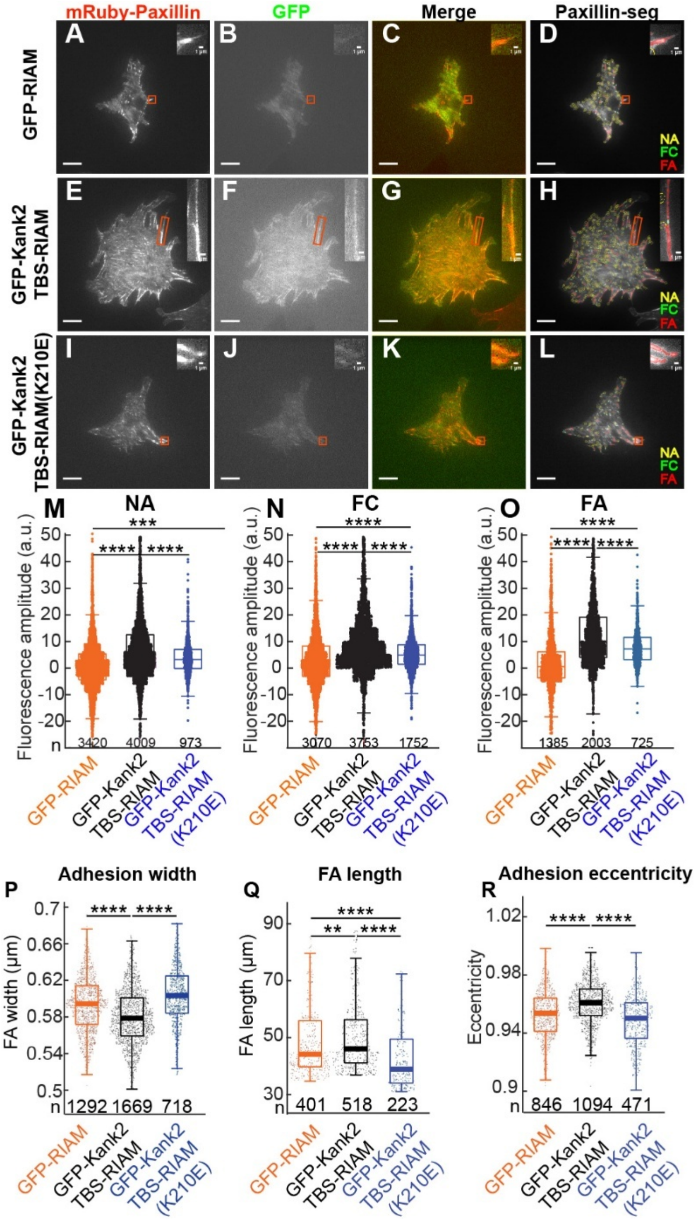

Figure S1. RIAM-Kank2TBS colocalizes with FA in a size-dependent manner and makes adhesions longer and thinner in a Rap1-dependent manner. (A-L) TIRF images of mRuby-paxillin **(A,E,I)**, GFP **(B,F,J)**, merge **(C,G,K)** and paxillin images with adhesion segmentation **(D,H,L)** of representative 3T3 fibroblasts expressing mRuby-paxillin and GFP-RIAM **(A-D)**, mRuby-paxillin GFP-RIAM Kank2TBS **(E-H)**, and mRuby-paxillin GFP-RIAM Kank2TBS (K210E) **(I-L)** on a 5kPa gel substrate. Scale bar: 20 µm. GFP-tagged RIAM and its variants were induced by 20 ng/ml of doxycycline. Adhesion segmentation shows NAs in yellow, focal complexes in green, and FAs in red. Inset: zoomed-in images displaying a representative FA in each red box. Inset scale bar: 1 µm; adhesion segmentation in FA (green), focal complex (red), NA (yellow). **(M-O)** Box plots of fluorescence intensity of NA (**M**), focal complex (FC, **N**) and FA (**O**) in GFP channel. **(P-R)** Box plot of FA width **(P)**, FA length **(Q)**, FA eccentricity **(R)**. The numbers shown on top of the x-axis are the numbers of adhesions collected from independently imaged cells, for GFP-RIAM (n=30), n=30 for GFP-RIAM Kank2TBS, and n=16 for GFP-RIAM Kank2TBS (K210E). **: $p<1x10^{-3}$, ***.: $p<1x10^{-5}$, ****.: $p<1x10^{-10}$ by Mann-Whitney U test.
